# Perception of cure among leprosy patients post completion of multi-drug therapy

**DOI:** 10.1186/s12879-021-06587-6

**Published:** 2021-09-06

**Authors:** Aleksandra Rosendo dos Santos, Pãmela Rodrigues de Souza Silva, Letícia Gomes Costa, Peter Steinmann, Eliane Ignotti

**Affiliations:** 1 Health Sciences Graduation Program, Federal University of Mato Grosso (UFMT), Cuiabá, Brazil; 2 Faculty of Health Sciences, University of the State of Mato Grosso (UNEMAT), Cáceres, Brazil; 3 Faculty of Nursing, Federal University of Mato Grosso (UFMT), Cuiabá, Brazil; 4 Swiss Tropical and Public Health Institute (Swiss TPH), Socinstrasse 57, 4051 Basel, Switzerland; 5 University of Basel, Basel, Switzerland

**Keywords:** Leprosy, Multidrug therapy, Disability, Cure, Brazil

## Abstract

**Background:**

Leprosy is a treatable disease; however, the release from treatment after completion of multidrug therapy (MDT) often does not equal absence of health problems. Consequently, sequelae interfere with the patient’s perception of cure. The objective of this study was to analyze the factors associated with the perception of not being healed among people treated for leprosy in a highly endemic area in Brazil.

**Method:**

A cross-sectional study of perceived cure of leprosy in the post-release from treatment period was conducted in Cáceres in the state of Mato Grosso, Brazil. The study included a total of 390 leprosy patients treated with MDT and released after completion of treatment from 1 January 2000 to 31 December 2017. The dependent variable was self-reported cure of leprosy; the independent variables included clinical, operational and socioeconomic variables.

**Results:**

Out of the 390 former leprosy patients, 304 (77.9%) perceived themselves as cured and 86 (22.1%) considered themselves unhealed. Among the latter, 49 (57.0%) reported muscle weakness and joint pains. Individuals with complaints related to leprosy post-release from treatment had a 4.6 times higher chance to self-report as unhealed (OR 4.6; 95% CI 2.5–8.5). Patients with physical disabilities (PD) grade 1 and 2 at the time of the study had a 3.1 (OR 3.1; 95% CI 1.3–7.4) and 8.8 (OR 7.7; 95% CI 3.5–21.9) times higher likelihood to self-identify as unhealed, respectively.

**Conclusion:**

Among successfully treated leprosy patients, a quarter self-report as unhealed of the disease. The factors associated with the perception of being unhealed are PD and complaints related to leprosy in the post-release from treatment phase.

**Supplementary Information:**

The online version contains supplementary material available at 10.1186/s12879-021-06587-6.

## Background

Newly diagnosed leprosy patients are treated with multidrug therapy (MDT). Globally, 16 million former leprosy patients have successfully completed MDT. According to the criteria of the World Health Organization (WHO), completion of the drug regimen is the only criterion for cure of leprosy [1]. However, completing leprosy treatment and being released may not represent the end of health problems related to the disease [2]. Studies have shown that for more than one third of the successfully treated patients, the physical disability (PD) grade worsens in the post-release from treatment period [3, 4]. This observation is relevant considering the limitations that PD can cause in people's lives, most notably impairments in the performance of daily activities [3], their tendency to predispose stigma, and the psychological, economic and social damage such feelings cause [5]. Brazil ranked second in the global number of leprosy cases in the year 2018 [6], and Mato Grosso was the most endemic among the Brazilian states in 2018 [7].

The symptoms related to PD can compromise the feeling of being cured of leprosy after completion of treatment with MDT [7]. For people affected by the disease, perceived cure may not only depend on the bacteriological and immunological status, but also the health condition at the end of treatment [8]. Generally, health reestablishment is characterized by release from treatment and return to daily work activities, family and social life [9] but these dimensions may be severely impacted even after MDT completion.

Moreover, a significant group of people do not believe in the cure of leprosy [10], even after treatment [11]. This fact deserves further investigation, due the need to identify the factors interfering with self-assessment as cured. In Brazil, in the post-release from treatment period, patients are excluded from the active leprosy registry as well as from the specific leprosy assistance program, regardless of the PD grade and other sequelae [12]. The recommended activities for preventing PD after treatment are the same as those during the treatment period. Thus, the main difference is in the availability and access to leprosy-specific health services. During treatment, people are accompanied in a systematic, regular and continuous manner while in the post-release period, they should seek assistance in the primary health care system according to their self-assessed needs [7].

PD includes chronic conditions requiring comprehensive care in the post-release from treatment period and still needs to be integrated effectively into the Brazilian health system [12]. The lack of continuous and qualified assistance for the management of sequelae of the disease may thus interfere with the self-perceived feeling of being cured from leprosy [8].

Given the number of people affected and disabled by leprosy, as well as the scarcity of information about the post-release from treatment period, it is relevant to analyze the factors associated with the perception of being cured or not among people treated for leprosy in the highly endemic areas in Brazil.

## Methods

### Study design and population

We conducted a cross-sectional study on the self-perception of cure from leprosy post-release from treatment in Cáceres in the state of Mato Grosso. The study population consisted of newly diagnosed leprosy patients who had completed treatment with MDT between 01 January 2000 and 31 December 2017. As the goal of MDT treatment is to cure leprosy, it is expected that the released patients self-perceive as healed after completing their treatment (the only established criterion for release after treatment) while patients may perceive the persistence of disabilities or pains as a sign of unsuccessful treatment and hence, not being cured.

### Data collection

This study was conducted within the project “Survival analysis: progression post-release from treatment of the physical disability grade of leprosy patients in a hyperendemic area of Brazil” [4]. We included only those patients resident in the urban area of Cáceres—all of them had been diagnosed and treated in the municipality and had a record of PD assessment at diagnosis and at release from treatment [4]. The address registered in the medical records and complementary information by health workers facilitated the identification of the current location of the participants. Potential study participants were considered “not located” after three unsuccessful home visits or unequivocal information that they had moved out of the study area. All participants read and signed an informed consent form authorizing the study data collection. After the participant's written consent, relevant data were obtained from medical records, through a semi-structured questionnaire and from a simplified neurological evaluation. The semi-structured questionnaire (Additional file 1) is part of a larger survey tool from the project mentioned above [4] and was prepared by a group of researchers and health workers specialized in leprosy. Health workers included one medical doctor, 3 registered nurses, 1 epidemiologist, 1 dentist, and 1 biochemist. The questionnaire was tested with 20 former leprosy patients from Cuiabá–Mato Grosso and revised. The main questions were: “Do you feel cured of leprosy?” If not: “Why not?” The fieldwork was carried out from January 2017 to February 2018. The field work was carried out by the 3 first authors, supported by the nurses and Community Health Workers of the primary care facilities of the municipality.

### Study variables

“Self-perception of cure (yes/no)” was defined as the main outcome. The factors considered as covariates in models to identify factors associated with self-perceived cure from leprosy were: gender (male, female); age group (0–15, 16–29, 30–59 and 60 + years); highest educational level (illiterate, elementary school, middle school, high school, university); household income (R$ 0–1000, R$ 1001–3000, R$3001–5000, R$ 5001 and more); social insurance (yes/no); having received a sick note (yes/no); detection mode (spontaneous [the patient directly presents to the health services], referral, other modes); leprosy classification (paucibacillary [PB] or multibacillary [MB]); leprosy reactions during treatment (yes/no) and post-release from treatment (yes/no); leprosy-related complaints during treatment (yes/no) and post-release from treatment (yes/no); and use of other medications during MDT treatment (yes/no).

The simplified neurological assessment was performed to determine the current PD grade pertaining to the eyes, hands and feet [7]. The assessment was performed in a health facility near the participants' residence, by trained health workers supported by a member of the research team with experience in neurological assessment of leprosy patients. The PD grade was classified as follows: grade 0—G0D (no leprosy-related PD), grade 1—G1D (decreased strength and/or loss of sensation) and grade 2—G2D (presence of visible disabilities and deformities) [7]. Individuals with open lesions requiring immediate care received assistance at home and, when necessary, were referred for care at the health center closest to their residence.

### Data analysis

The Chi-square or Fisher's exact tests were used to evaluate the difference between proportions of covariates for the individuals who self-perceived as cured and as unhealed, respectively.

Logistic stepwise regression was used to test the independent association of covariates with the self-perception as cured or unhealed. The magnitude of the association was estimated using odds ratios (OR). To adjust the regression model, two groups stratified with respect to leprosy treatment time were considered for analysis. One group included events during treatment with MDT and the other group covered the post-release from treatment with MDT period. For the first group we analyzed gender, social insurance, leprosy classification, leprosy reaction, complaints during treatment, other medications and having received a sick note during treatment; for the second group we analyzed gender, leprosy reactions and complaints post-release from treatment and the PD grade post-release from treatment. The set of variables in each group was analyzed independently from that in the other group. The model included variables reaching in the initial analysis a significance level of p < 0.2. The final model was tested at a significance level of p < 0.05. There were no missing data, and all analyses were performed using SPSS 21.0 (SPSS Inc. Chicago, IL).

### Ethical considerations

The study was approved by the Ethics Committee of the University Hospital Júlio Müller (protocol number 555 567; 02/26/2014). A STROBE checklist is available as (Additional file 2).

## Results

**Fig. 1:**
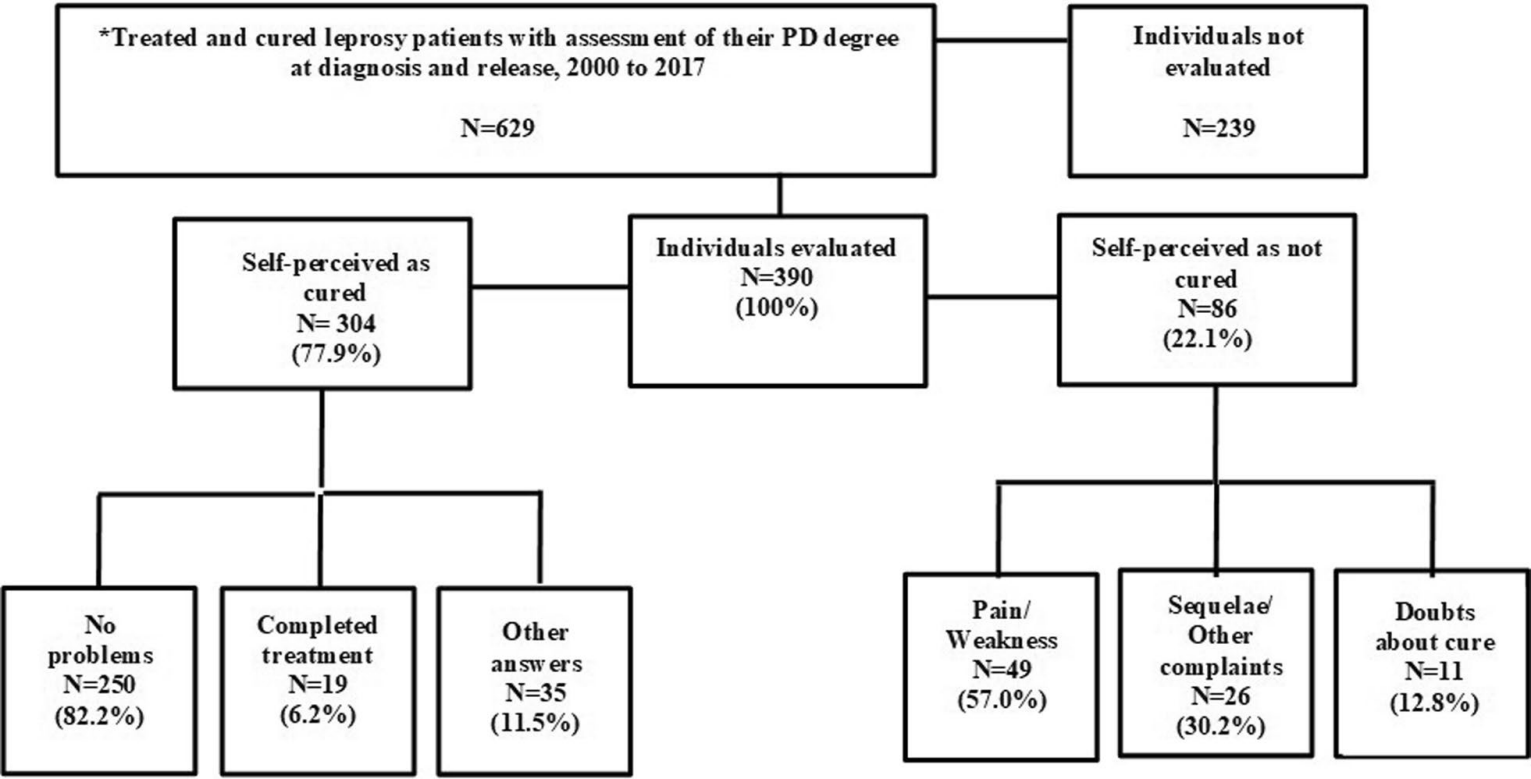
Study flowchart of leprosy patients from Cáceres–Mato Grosso, Brazil released from treatment 2000–2017, stratified by recruitment status and self-perceived cure

A total of 390 individuals participated in the study. Among them, 77.9% (n = 304) reported being cured and 22.1% (n = 86) felt unhealed. Among those who self-perceived as cured, 82.2% (n = 250) associated this self-assessment with the absence of signs and symptoms of the disease. For 57.0% (n = 49) of those declaring themselves unhealed, the main reasons were joint pains and muscle weakness in the post-release from treatment phase. Another 239 potential study participants could not be included. Among them, 65 had moved to another city, 40 had died and 2 refused to participate in the study (Fig. 1). With regard to age and gender, no statistically significant difference was observed between potential and actual study participants.

Table 1 shows that the proportion of former leprosy patients who perceived themselves as cured declined significantly with increasing PD grade as determined at the time of the study (p < 0.01).

**Table 1:** Self-perceived cure from leprosy and physical disability (PD) grade at the time of the study among leprosy patients released from treatment as cured, Cáceres–Mato Grosso, Brazil

PD grade	Self-perceived as cured		Self-perceived as not cured		x^2^	p value
	n	%	n	%		
Grade 0	127	94.8	7	5.2	1	–
Grade 1	132	78.6	36	21.4	16.0	0.001
Grade 2	45	51.1	43	48.9	57.9	0.001

Significant differences between the individuals who perceived themselves as cured compared to those who considered themselves unhealed were identified only for the variables related to social insurance (Table 2).

**Table 2:** Self-perceived cure from leprosy among leprosy patients released from treatment as cured, stratified by demographic and socio-economic characteristics; Cáceres–Mato Grosso, Brazil

Variables	Self-perceived as cured		Self-perceived as not cured		x^2^	p value
	n	%	n	%		
Age group (years)						
0–15	2	66.7	1	33.3	1	–
16–29	16	94.1	1	5.9	2.13	0.143
30–59	176	76.2	55	23.8	0.14	0.561
60 +	110	79.1	29	20.9	0.27	0.600
Gender						
Male	152	74.1	53	25.9	3.63	0.066
Female	152	82.2	33	17.8	1	–
Educational level						
Illiterate	44	78.6	12	21.4	0.021	0.884
Elementary School	82	72.6	31	27.4	0.586	0.443
Middle School	88	78.6	24	21.4	0.025	0.874
High School	70	83.3	14	16.7	0.148	0.699
University	20	80.0	5	20.0	1	–
Household incomes (R$)						
R$ 0–1,000	90	72.0	35	28.0	3.01	0.082
R$ 1100–3000	149	79.3	39	20.7	1.03	0.308
R$ 3100–5000	38	82.6	08	17.4	0.28	0.594
R$ 5000 +	27	87.1	04	12.9	1	–
Social insurance						
Yes	26	49.1	27	50.9	1	–
No	278	82.5	59	17.5	29.78	0.001

Table 3 shows that there are significant differences between former leprosy patients who perceived themselves as cured and those who self-perceived as unhealed with regard to leprosy classification, leprosy-related complaints during treatment, leprosy reactions during the treatment period and post-release from treatment, use of other medications during MDT treatment and having received a sick note. The mode of detection and leprosy classification were not associated with self-perceived cure.

**Table 3:** Self-perceived cure among leprosy patients released from treatment as cured, stratified by operational and clinical characteristics; Cáceres–Mato Grosso, Brazil

Variables	Self-perceived as cured		Self-perceived as not cured		x^2^	p value
	n	%	n	%		
Detection mode						
Spontaneous	214	77.0	64	23.0	0.330	0.565
Referral	56	80.0	14	20.0	0.015	0.902
Other modes	34	81.0	8	19.0	1	–
Leprosy classification						
Paucibacillary	205	81.0	48	19.0	1	–
Multibacillary	99	72.3	38	27.7	3.97	0.046
Complaints during treatment						
Yes	171	71.0	70	29.0	17.96	0.001
No	133	89.3	16	10.7	1	–
Leprosy reaction during treatment						
Yes	70	59.3	48	40.7	34.15	0.001
No	234	80.3	38	14	1	–
Other medications						
Yes	79	62.2	48	37.8	27.15	0.001
No	225	85.5	38	14.5	1	–
Complaints post-release from treatment						
Yes	101	59.8	68	40.2	57.38	0.001
No	203	91.9	18	8.1	1	
Sick note during treatment						
Yes	30	55.6	24	44.4	15.23	< 0.001
No	274	81.5	62	18.5	1	–
Leprosy reactions post-release from treatment						
Yes	59	57.3	44	42.7	34.78	< 0.001
No	245	85.4	42	14.6	1	–

The variables that were independently associated with self-perceived cure from leprosy are shown in Table 4. Former patients who had received a social insurance payment during the treatment phase were 3 time more likely to self-perceive as unhealed compared to those who had never received any social insurance grants (adjusted OR 3.07; 95% CI 1.31–7.19). Those presenting leprosy reactions during the treatment were 2.6 times more likely to self-perceive as unhealed (adjusted OR 2.60; 95% CI 1.36–4.97) than those reporting complaints (adjusted OR 2.12; 95% CI 1.11–4.04) or taking other medications beyond MDT (adjusted OR 2.12; 95% CI 1.16–3.87). Individuals with complaints post-release from leprosy treatment were 4.6 times more likely to self-perceive as unhealed (adjusted OR 4.62; 95% CI 2.51–8.48), and if presenting leprosy reactions post-release from treatment, they were also more likely to self-perceive as unhealed (adjusted OR 2.82; 95% CI 1.62–4.88). Similarly, those who were evaluated with PD grade 1 and 2 at the time of the study were 3.0 (adjusted OR 3.07; 95% CI 1.27–7.41) and 8.8 (adjusted OR 8.78; 95% CI 3.52–21.92) times more likely to perceive themselves as unhealed, respectively.

**Table 4:** Logistic regression analysis of self-perceived leprosy cure post-release from treatment with socio-economic, operational and clinical variables stratified by MDT-related timing; Cáceres–Mato Grosso, Brazil

Variable	Self-perceived as cured		Self-perceived as unhealed		Crude OR		Adjusted OR	
	N	%	N	%	(95% IC)	p value	(95% IC)	p value
During treatment with MDT^a^								
Social Insurance								
Yes	26	49.1	27	50.9	4.89 (2.66–8.98)	0.001	3.07 (1.31–7.19)	0.010
No	278	82.5	59	17.5	1		1	
Leprosy classification								
Paucibacillary	205	81.0	48	19.0	1		1	
Multibacillary	99	72.3	38	27.7	1.63 (1.00–2.67)	0.047	0.88 (0.49–1.58)	0.684
Leprosy reaction during treatment								
Yes	70	59.3	48	40.7	4.22 (2.55–6.97)	0.001	2.60 (1.36–4.97)	0.004
No	234	80.3	38	14.0	1		1	
Complaints during treatment								
Yes	171	71.0	70	29.0	3.40 (1.88–6.12)	0.001	2.12 (1.11–4.04)	0.021
No	133	89.3	16	10.7	1		1	
Sick note during treatment								
Yes	30	55.6	24	44.4	3.68 (2.00–6.75)	0.001	0.89 (0.36–2.18)	0.801
No	274	81.5	62	18.5	1		1	
Other medication								
Yes	79	62.2	48	37.8	3.78 (2.28–6.24)	0.001	2.12 (1.16–3.87)	0.015
No	225	85.5	38	14.5	1		1	
Post-release from treatment with MDT^b^								
Complaints post-release from treatment								
Yes	101	59.8	68	40.2	7.59 (4.28–13.44)	0.001	4.62 (2.51–8.48)	0.001
No	203	91.9	18	08.1	1		1	
Leprosy reactions post-release from treatment^d^								
Yes	59	57.3	44	42.7	4.35 (2.61–7.24)	0.001	2.82 (1.62–4.88)	0.001
No	245	85.4	42	14.6	1		1	
PD grade post-release from treatment								
Grade 0	127	94.8	07	5.2	1		1	
Grade 1	132	78.6	36	21.4	4.94 (2.12–11.52)	0.001	3.07 (1.27–7.41)	0.012
Grade 2	45	51.1	43	48.9	17.33(7.27–41.30)	0.001	8.78 (3.52–21.92)	0.001

*OR* odds ratio; *CI* confidence interval

^a^Adjusted by the variables related the period during treatment

^b^Adjusted by the variables related the period post-release from treatment

Gender and age groups were explored as subgroups for the variables associated with the outcome, with no additional significant findings. We didn’t find interactions between the study variables.

## Discussion

To our knowledge, this is the first study that investigated the self-perceived outcome of leprosy treatment in the period post-release from MDT treatment in a high endemicity area. About a quarter of the study participants considered themselves as unhealed, with joint pain and muscle weakness being the main reasons for this self-assessment. Among those who self-perceived as cured, the majority justified their assessment by not having signs and symptoms of the disease. Thus, complaints and leprosy reactions during and post-release from treatment were central to the perception of cure. Indeed, former patients with PD grade 1 and 2 were much more likely to self-report as unhealed in the period post-release from treatment than their peers without PD. Social insurance payments are provided by the government only for those patients who cannot work for some months, or permanently [13] as a consequence of leprosy complications. It thus distinguishes a group of individuals presenting more clinical and social vulnerabilities.

In line with global guidelines, Brazil uses only one criterion for release from leprosy treatment: the completion of standard treatment with MDT [7]. Treatment is bactericidal and bacteriostatic for *M. leprae* but does not result in recovery from neural damage and does not prevent the onset of disease complications [14]. Studies have shown that PD [2] and reactions [2, 3, 15] frequently occur during the post-release from treatment period [4]. This is particularly troubling because the period post-release from treatment is not part of the active disease progression surveillance and individuals are excluded from the specific leprosy assistance program providing regular and systematic care [7]. Of note, the occurrence of true relapses among those feeling unhealed is unlikely since quality leprosy diagnosis and management is easily accessible and free in the study area.

In the 1990s, after the adoption of the MDT treatment protocol, a study carried out in Brazil showed that about 30% of the treated patients did not believe in actual cure of the disease [16]. Another study carried out in India, with cured individuals, shows that about 69% of the patients had been afraid when diagnosed with leprosy, a higher frequency when compared to conditions such as cancer, AIDS and diabetes. For 63% of the participants, the reason for their fear was the possibility of disabilities and discrimination related to the disease, while only 20% believed in the possibility of real cure [11].

Joint pains and muscle weakness are reported by more than half of all former patients who perceived themselves as unhealed post-release from leprosy treatment. The development of leprosy is generally painless and characterized mainly by numbness and/or anesthesia in certain areas of the body [7]. However, nerve damage, the main cause of PD [16] when not diagnosed and treated early [7], is one of the main causes of pain in people affected by leprosy. We know also that leprosy patients have less muscle strength [17]. A study in India has shown that 60% of people cured of leprosy experience some type of pain [18]. The most common is neuropathic pain that can occur late [19]. A study in Nepal had shown that around 70% of the patients treated with MDT have symptoms of neuropathic pain [20]. In addition, the study suggested that the presence of neuropathic pain may be indicative of leprosy complications, and 70% of the patients have had or still had reactions at the time of the assessment, and 43% were classified as having grade 2 disability, suggesting considerable interference in carrying out daily activities [20]. Although common among people affected by leprosy, neuropathic pain is difficult to diagnose in almost 50% of cases due to the absence of an appropriate protocol, general practitioners being unfamiliar with the condition and its assessment, and treatments that can mask the condition. Indeed, individuals with sequelae who reported feeling pain have twice the risk of developing limitations related to the performance of daily activities [13, 21]. For this reason, adequate assistance for these complications including biopsychosocial rehabilitation is important as these patients are more susceptible to interferences in daily life and psychological suffering than their peers without neuropathic pain [20].

The multivariate analysis showed that the current PD grade is independently associated with self-perceived persistence of the disease in the period post-release from treatment; the higher the PD grade the greater the chance of self-reporting as unhealed. This finding shows that the definition of cure for leprosy may need to be revised to include indicators beyond the completion of MDT.

Former patients who have reported signs or symptoms of complications are almost 4 times more likely to self-report as unhealed compared to their peers without such complaints. Therefore, it seems necessary to consider that the successful completion of MDT and release from treatment as cured may not represent the end of all health problems related to the disease [2]. For some patients, leprosy has become a curable disease due to MDT while for others, it is a chronic pathology for which a treatment may be available but not cure [8].

In addition to the size of the group that self-perceived as unhealed even post-release from leprosy treatment, the percentage of study participants that self-perceived as cured but presented PD in the post-release from treatment phase is important: 43.4% had grade 1 and 14.8% grade 2 disability, with many individuals not realizing their physical disabilities. The lack of awareness for their real condition can hamper self-control to limit activities, worsening PD [5, 21].

A limitation to our study is the inability to attribute causality to certain observations. Of note, complaints related to leprosy post-release from treatment could either be the reason or the consequence of the (perceived) persistence of the disease. We decided to maintain this variable in our study to highlight the importance of, and demand for, social and medical assistance among leprosy patients. Great care was taken at the time of the interview to avoid recall bias. Additionally, the importance of the disease in the individual’s life also contributes to a reduction in memory bias.

The characteristics of the study site and health services in the study area are comparable to those of other endemic municipalities in Brazil. We therefore belief that our results are valid beyond the study area. Our results also confirm other studies that call for care for former leprosy patients in the post-release from treatment period [4]. PD prevention and rehabilitation through the provision of qualified services seems important to promote the feeling of cure [22, 23].

## Conclusions

Among former leprosy patients released from treatment, one quarter perceived themselves as not cured of the disease. The factors associated with this perception include health complaints, leprosy reactions and receiving social insurance grants during and post release from treatment. Joint pains and muscle weakness are the main reasons why people treated for leprosy perceived themselves as unhealed even after completion of treatment. There is a considerable need to offer continued attention to former patients who have ended their treatment with MDT.

## Supplementary Information

**Additional file 1:** Santos_Questionnaire Perception of leprosy cureR3

**Additional file 2:** Santos_STROBE checklist

## Data Availability

The datasets used and/or analyzed during the current study are available from the corresponding author on reasonable request.

## Abbreviations

G0D: Grade 0 disability
G1D: Grade 1 disability
G2D: Grade 2 disability
CI: Confidence interval
MDT: Multidrug therapy
PD: Physical disability
WHO: World Health Organization
