# Supplementary material for: Perception of cure among leprosy patients post completion of multi-drug therapy
Source: BMC Infect Dis. 2021 Sep 6;21:916. doi: 10.1186/s12879-021-06587-6 (PMC8419967; doi:10.1186/s12879-021-06587-6)
Supplement: Supplementary file 1 — Additional file 1: Santos_Questionnaire Perception of leprosy cureR3 [file 12879_2021_6587_MOESM1_ESM.docx]

**Information collected in the Medical Record**

| **Project registration number:** | | **Medical record number:** | | **Date of collection:**  **_____/_____/_____** |
| --- | --- | --- | --- | --- |
| **General data** | | | | |
| **Patient's name:**  **Zone: 1.urban 2.rural**  **Unit: 1.Good Samaritan 2.PSF** | | | | |
| **Patient Identification** | | | | |
| **Q 1** | **Sex** | |  | |
| **Q 2** | **Date of birth** | |  | |
| **Q 3** | **Race** | |  | |
| **Q 4** | **Education** | |  | |
| **Q 05** | **Diagnosis Occupation** | |  | |

| **Clinical and Epidemiological Characteristics of Leprosy** | | | | | | | |
| --- | --- | --- | --- | --- | --- | --- | --- |
| **Q 06** | **Input mode** | |  | | | | |
| **Q 07** | **New case detection mode** | |  | | | | |
| **Q 08** | **Number of skin lesions** | |  | | | | |
| **Q 09** | **Clinical Form (Madrid)** | |  | | | | |
| **Q 10** | **Operational Classification** | |  | | | | |
| **Q 11** | **Number of nerves affected in the diagnosis** | |  | | | | |
| **Q 12** | **Bacilloscopy** | |  | | | | |
| **Q 13** | **Therapeutic scheme** | | **1.PQT/PB/6 months**  **2.PQT/MB /12 months**  **3.Other substitute schemes** | | | | |
| **Q 14** | **Date of diagnosis** | | **_____/_____/_____** | | | | |
| **Q 15** | **MDT start date** | | **_____/_____/_____** | | | | |
| **Q 16** | **End date of MDT** | | **_____/_____/_____** | | | | |
| **Q 17** | **Release date due to cure** | | **_____/_____/_____** | | | | |
| **Q 18** | **Start date of signs and symptoms** | |  | | | | |
| **Q 19** | **Leprosy reaction during treatment?** | | **1. Yes**  **2. No**  **9. Do not know** | | | | **Reaction type:**  **1.Type I 2.TypeII 3.Neuritis**  **4. had no reaction**  **9. Do not know** |
| **Q 20** | **Leprosy reaction after release due to cure?** | | 1. **Yes 2. No**   **9. Do not know** | | | | **Reaction type:**  **1.Type I 2.TypeII 3.Neuritis**  **4. had no reaction**  **9. Do not know** |
| **Q 21** | **Did you receive a medical certificate for treatment?**  **1. Yes 2. No 9.Do not Know** | | | | **Date of the certificate:**  **____/____/_____** | **Clearance time**  **___________________** | |
| **Q 22** | **Received medical certificate after release for cure?**  **1. Yes 2. No 9.Do not Know** | | | | **Date of the certificate:**  **__/____/_____** | **Clearance time**  **___________________** | |
| **Q23** | **Did you rest during the treatment?**  **1. Yes 2. No 9.Do not Know** | | | | **In what location?** | | |
| **Q24** | **Complaints during treatment?**  **1. Yes 2. No 9.Do not Know** | | | | **Which are?** | | |
| **Q25** | **Were laboratory tests requested?**  **1. Yes 2. No 9.Do not Know** | | | | **Which are?** | | |
| **Q26** | **Laboratory changes?**  **1. Yes 2. No 9.Do not Know** | | | | **Which are?** | | |
| **Q27** | **Change in medication regimen?**  **1. Yes 2. No 9.Do not Know** | | | | **Which are?** | | |
| **Q28** | **Other medications prescribed?**  **1. Yes 2. No 9.Do not Know** | | | | **Which are?** | | |
| **Q29** | **Post-release complaints from treatment?**  **1. Yes 2. No 9.Do not Know** | | | | **Which are?** | | |
| **Q30** | **Contact exams?**  **1. Yes 2. No 9.Do not Know** | | | | **How many examined** | | |
|  |  |  |  |  |  |  | |
| **Q34** | **1. Yes 2. No 9.Do not Know** | | | | **Kinship** | | |
| **Assessment of the Degree of Physical Disability** | | | | | | | |
| **Q35** | **Degree of disability in diagnosis** | **1. Grade 0 2. Grade 1 3. Grade 2 4. Not rated**  **Date of the certificate: _____/_____/_____** | | | | | |
| **Q 36** | **Degree of disability at release from treatment** | | | **1. Grade 0 2. Grade 1 3. Grade 2 4. Not rated**  **Date of the certificate _____/_____/_____** | | | |

#

# Information collected with the Patient (Interview)

| ***Interview date: _____/_____/_____*** | | | | |  |
| --- | --- | --- | --- | --- | --- |
| ***Income Conditions*** | | | | |  |
| **Q 37** | | **Marital Status** | |  |  |
| **Q 38** | | **Current schooling** | |  | |
| **Q 39** | | **Do you perform a paid activity after the occurrence of the disease?** | | 1. **Yes** 2. **No**   **Which one:_________________________** |  |
| **Q 40** | | **How many people live with you?** | | **Number: ___________________________** |  |
| **Q 41** | | **Family income** | | 1. **no income R$____________________** |  |
| **Q 42** | | **Do you usually drink alcohol?** | | 1. **Yes 3. Do not drink** 2. **No** |  |
| **Q 44** | | **How much money do you make in a month?** | | 1. **No income** 2. **R$ ____________________** |  |
| **Q 45** | | **Did you have to change of services after the disease occurrence?** | | 1. **Yes** 2. **No**   **What was your occupation:____________** |  |
| **Q 46** | | **Do you started to receive some government benefit after the disease occurrence?** | | 1. **Yes** 2. **No**   **Which one:________________________** |  |
| **Q 47** | | **Do you feel guilty (upset with yourself) for the way you usually drink?** | | 1. **Yes** 2. **No** |  |
| ***Clinical and Epidemiological Characteristics of Leprosy*** | | | | |  |
| **Q 48** | **When the symptoms of the disease started?** | | **Approximate time:__________________** | |  |
| **Q 49** | **Time between onset of symptoms and start of treatment** | | **Approximate time: _________________** | |  |
| **Q 50** | **Have you ever felt that you should decrease the amount of drink or stop drinking?** | | 1. **Yes 3. Do not drink** 2. **No** | |  |
| **Q 51** | **Did you have leprosy reactions during treatment?** | | 1. **Yes** 2. **No 3. Do not know** | |  |
| **Q 52** | **Did you were remove from work during the period of leprosy reactions in the treatment?** | | 1. **Yes** 2. **No**   **For how long:____________________** | |  |
| **Q 53** | **Did you have leprosy reactions after release from MDT?** | | 1. **Yes** 2. **No 3. Do not know** | |  |
| **Q 54** | **Did you were remove from work during the period of leprosy reactions after release from MDT?** | | 1. **Yes** 2. **No**   **For how long: ___________________** | |  |
| **Q 55** | **Did you receive orientation on preventing disability at release?** | | 1. **Yes** 2. **No** | |  |
| **Q 56** | **What orientation did you receive at release?** | | 1. **Inspection of the eyes for redness** 2. **Orientation of the importance of blinking the eyes frequently** 3. **Wearing sunglasses** 4. **Use of eye drops** 5. **Inspection of hands and feet for signs of wounds** 6. **Hydration and lubrication** 7. **Dressings** 8. **Plastered splints** 9. **Shoes modification** 10. **Use of a dorsiflexor device** 11. **Resting** 12. **Performing exercises** | |  |
| **Q 57** | **Do people annoy you because they criticize the way you are drinking?** | | 1. **Yes 3. Do not know** 2. **No** | |  |
| **Q 58** | **Did you experience any of the following symptoms after being released for a cure:** | | 1. **Dry / stuffy / bleeding nose** 2. **Red / dry / itchy / sore eyes** 3. **Pain in the limbs** 4. **Weakness in the limbs** 5. **Tingling** 6. **Losing the slipper** 7. **Have / had been injured** | |  |
| **Q 59** | **When symptoms of complications started after release from MDT?** | | **Time in months approximately:________** | |  |
| **Q60** | **Did you have any problem during the treatment?**  **1. Yes 2. No** | | **Which problems?** | |  |
| **Q61** | **Were requested laboratory tests? 1. Yes 2. No** | | **Which one?** | |  |
| **Q62** | **Did you have problems in the exams?**  **1. Yes 2. No** | | **Which one?** | |  |
| **Q63** | **Did you have to change your medicine?**  **1. Yes 2. No** | | **Which one?** | |  |
| **Q64** | **Did you receive another medicines?**  **1. Yes 2. No** | | **Which one?** | |  |
| **Q65** | **Did you have problems after de release from the treatment?**  **1. Yes 2. No**  **Date:** | | **What post-release problems?** | |  |
| **Q66** | **Were your relatives examined?**  **1. Yes 2. No** | | **How many relatives were examined?**  **How many relatives were not examined?** | |  |
| **Q67** | **Did the parents have the presence of vaccine scar (BCG)?**  **1. Yes 2. No** | | **How many had the scar?**  **How many did not have the scar?** | |  |
| **Q68** | **How many with one scars?** | |  | |  |
| **Q69** | **How many with two scars?** | |  | |  |
| **Q70** | **Did the contacts have leprosy?**  **1. Yes 2. No** | | **Degree of kinship:______________________** | |  |
| **Q71** | **What does mean cure for you?** | |  | |  |
|  |  | |  | |  |
|  |  | |  | |  |
| **Q72** | **Do you feel cured of leprosy?** | | **1. Yes 2. No** | |  |
|  | **Why?** | |  | |  |
|  |  | |  | |  |

**Physical Evaluation of the Patient**

**Assessment of Disability Degree in Post-release due to Cure**

**DATE: _____/_____/_____ Name of the Pacient:__________________________**

**Birthday: ___/____/____ Age in the date of evaluation:______**

| **Inspection and assessment of sensitivity in hands and feet** | | | | | | | | |
| --- | --- | --- | --- | --- | --- | --- | --- | --- |
| **Right Hand**  **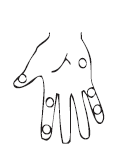** | | | **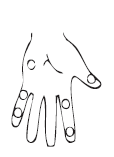Left Hand** | | **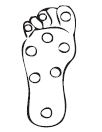Right Foot** | | | **Left Foot**  **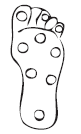** |
| **R** | **L** | **Number of nerves affected** | | **R** | | **L** | **Number of nerves affected** | |
|  |  | 1. **None nerve affected** | |  | |  | 1. **None nerve affected** | |
|  |  | 1. **One nerve affected** | |  | |  | 1. **One nerve affected** | |
|  |  | 1. **Two nerves affected** | |  | |  | 1. **Two nerves affected** | |
|  |  |  | |  | |  | 1. **Three nerves affected** | |

**Classification of the Grade of Physical Disability**

| **Grade** | **Eyes** | | | | | **Hand** | | | | **Foot** | | | |
| --- | --- | --- | --- | --- | --- | --- | --- | --- | --- | --- | --- | --- | --- |
|  | **Signs and / or symptoms** | **R** | | **L** | **Signs and / or symptoms** | | **R** | **L** | **Signs and / or symptoms** | | **R** | **L** |  |
| **0** | **No problem with the eyes** |  | |  | **No problem with the hand** | |  |  | **No problem with the feet** | |  |  |  |
| **1** | **Decrease or loss of sensation** |  | |  | **Decrease or loss of sensation (do not fell 2g)** | |  |  | **Decrease or loss of sensation (do not feel 2g)** | |  |  |  |
| **2** | **Logophthalmos and / or ectropion** |  | |  | **Trophic injuries and / or traumatic injuries** | |  |  | **Trophic injuries and / or traumatic injuries** | |  |  |  |
|  | **Trichiasis** |  | |  | **Claws** | |  |  | **Claws** | |  |  |  |
|  | **Central corneal opacity** |  | |  | **Reabsorption** | |  |  | **Reabsorption** | |  |  |  |
|  | **Visual acuity less than 0.1 or do not count fingers in a distance of 6 meters** |  | |  | **Fallen hand** | |  |  | **Fallen foot** | |  |  |  |
|  |  |  |  |  |  |  |  |  | **Ankle contracture** | |  |  |  |
|  | **Highest grade** |  | |  | **Highest grade** | |  |  | **Highest grade** | |  |  |  |
| **Grade of Physical Disability** | | | 1. **Grade 0 2. Grade 1 3. Grade 2** | | | | | | | | | | |
