# Supplementary material for: Perception of cure among leprosy patients post completion of multi-drug therapy
Source: BMC Infect Dis. 2021 Sep 6;21:916. doi: 10.1186/s12879-021-06587-6 (PMC8419967; doi:10.1186/s12879-021-06587-6)
Supplement: Supplementary file 2 — Additional file 2: Santos_STROBE checklist [file 12879_2021_6587_MOESM2_ESM.doc]

STROBE Statement—Checklist of items that should be included in reports of ***cross-sectional studies***

|  | Item No | Recommendation |
| --- | --- | --- |
| **Title and abstract** | 1 | (*a*) Indicate the study’s design with a commonly used term in the title or the abstract  Page number 2, Abstract, line 26-28 |
|  |  | (*b*) Provide in the abstract an informative and balanced summary of what was done and what was found  Page number 2, Abstract, line 26-37 |
| Introduction | | |
| Background/rationale | 2 | Explain the scientific background and rationale for the investigation being reported  Page number 3, Background, line 46-76 |
| Objectives | 3 | State specific objectives, including any prespecified hypotheses  Page number 4, Background, line 77-80 |
| Methods | | |
| Study design | 4 | Present key elements of study design early in the paper  Page number 4, Methods, Study Design and Population, line 84-85; 87-91 |
| Setting | 5 | Describe the setting, locations, and relevant dates, including periods of recruitment, exposure, follow-up, and data collection  Page number 4, Methods, Study Design and Population, line 85-91  Page number 5, Methods, Study Design and Population, line 110 |
| Participants | 6 | *(*a) Give the eligibility criteria, and the sources and methods of selection of participants  Page number 4, Methods, Study Design and Population, line 85-87  Page number 5, Data Collection, line 96-101 |
| Variables | 7 | Clearly define all outcomes, exposures, predictors, potential confounders, and effect modifiers. Give diagnostic criteria, if applicable  Main outcome: Page number 5, Methods, Study Variables, line 115  Covariates (predictors): Page number 6, Methods, Study Variables, line 115-134  Diagnostic criteria: N/A |
| Data sources/ measurement | 8* | For each variable of interest, give sources of data and details of methods of assessment (measurement). Describe comparability of assessment methods if there is more than one group  Self-perception of cure (yes/no) and predictors: Page number 5, Methods, Data Collection, line 109-112.  Physical disability (PD) grade: Page number 6, Methods, Study Variables, line 126-134 |
| Bias | 9 | Describe any efforts to address potential sources of bias  Page number 5, Methods, Data Collection, line 96-98  Page number 6, Methods, Study Variables, line 126-134  Page number , Discussion, line 288–294. |
| Study size | 10 | Explain how the study size was arrived at  Not applicable. In this study, we work with the population and not just a sample.  Page number 5, Methods, Data Collection, line 96-98 |
| Quantitative variables | 11 | Explain how quantitative variables were handled in the analyses. If applicable, describe which groupings were chosen and why  Page number 6 and 7, Methods, Data Analysis, line 137-152 |
| Statistical methods | 12 | *(*a) Describe all statistical methods, including those used to control for confounding  Page number 7, Methods, Data Analysis, line 140-152 |
|  |  | (*b*) Describe any methods used to examine subgroups and interactions  Page number 7, Methods, Data Analysis, line 140-152.  Page number 10, Results, line 218–220. |
|  |  | (*c*) Explain how missing data were addressed  Absence of missing data in the study.  Page number 7, Methods, Data Analysis, line 151-152 |
|  |  | (*d*) If applicable, describe analytical methods taking account of sampling strategy  N/A |
|  |  | (*e*) Describe any sensitivity analyses  N/A |
| Results | | |
| Participants | 13* | (a) Report numbers of individuals at each stage of study—eg numbers potentially eligible, examined for eligibility, confirmed eligible, included in the study, completing follow-up, and analysed  Page number 7, Results, line 159-167  Page number 9, Results, Figure 1 |
|  |  | (b) Give reasons for non-participation at each stage  Page number 7, Results, line 163-167 |
|  |  | (c) Consider use of a flow diagram  Page number 9, Results, Figure 1 |
| Descriptive data | 14* | (a) Give characteristics of study participants (eg demographic, clinical, social) and information on exposures and potential confounders  Page number 10, Results, line 186-200  Table 1: page number 24  Table 2: page number 25-26  Table 3: page number 27-28 |
|  |  | (b) Indicate number of participants with missing data for each variable of interest  N/A |
| Outcome data | 15* | Report numbers of outcome events or summary measures  Table 1: page number 24  Table 2: page number 25-26  Table 3: page number 27-28 |
| Main results | 16 | *(*a) Give unadjusted estimates and, if applicable, confounder-adjusted estimates and their precision (eg, 95% confidence interval). Make clear which confounders were adjusted for and why they were included  Page number 10, Results, line 202-215  Table 4: page number 29-31 |
|  |  | (*b*) Report category boundaries when continuous variables were categorized  Page number 5-6, Methods, Study Variables, line 117-119  Page number 6, Methods, Study Variables, line 129-132  Table 1: page number 24  Table 2: page number 25-26  Table 3: page number 27-28 |
|  |  | (*c*) If relevant, consider translating estimates of relative risk into absolute risk for a meaningful time period  N/A |
| Other analyses | 17 | Report other analyses done—eg analyses of subgroups and interactions, and sensitivity analyses  N/A |
| Discussion | | |
| Key results | 18 | Summarise key results with reference to study objectives  Page number 11-14, Discussion, line 222-287 |
| Limitations | 19 | Discuss limitations of the study, taking into account sources of potential bias or imprecision. Discuss both direction and magnitude of any potential bias  Page number 14, Discussion, line 288-294 |
| Interpretation | 20 | Give a cautious overall interpretation of results considering objectives, limitations, multiplicity of analyses, results from similar studies, and other relevant evidence  Page number 14, Discussion, line 295-299  Page number 14, Conclusions, line 302-308 |
| Generalisability | 21 | Discuss the generalisability (external validity) of the study results  Page number 14, Discussion, line 295-299 |
| Other information | | |
| Funding | 22 | Give the source of funding and the role of the funders for the present study and, if applicable, for the original study on which the present article is based  Page number 17-18, Funding, line 337-341 |

*Give information separately for exposed and unexposed groups.

**Note:** An Explanation and Elaboration article discusses each checklist item and gives methodological background and published examples of transparent reporting. The STROBE checklist is best used in conjunction with this article (freely available on the Web sites of PLoS Medicine at http://www.plosmedicine.org/, Annals of Internal Medicine at http://www.annals.org/, and Epidemiology at http://www.epidem.com/). Information on the STROBE Initiative is available at www.strobe-statement.org.
